# No Major Nerve Regeneration Seems to Occur during Recovery of Ulnar Neuropathy at the Elbow

**DOI:** 10.3390/jcm12123906

**Published:** 2023-06-07

**Authors:** Simon Podnar

**Affiliations:** Institute of Clinical Neurophysiology, Division of Neurology, University Medical Center Ljubljana, SI-1525 Ljubljana, Slovenia; simon.podnar@kclj.si

**Keywords:** electrodiagnosis, focal neuropathy, motor unit potential, reinnervation, ulnar neuropathy

## Abstract

**Introduction:** There are three main potential mechanisms of recovery after nerve lesion: (1) resolution of conduction block, (2) collateral reinnervation, and (3) nerve regeneration. Their relative contributions in recovery after focal neuropathies are not well established. **Methods:** In a group of previously reported prospective cohort of patients with ulnar neuropathy at the elbow (UNE), I performed a post-hoc analysis of their clinical and electrodiagnostic findings. I compared amplitudes of the compound muscle action potential (CMAP) and sensory nerve action potential (SNAP) on ulnar nerve stimulation, as well as qualitative concentric needle electromyography (EMG) findings in the abductor digiti minimi muscle on the initial and follow-up examinations several years later. **Results:** Altogether, 111 UNE patients (114 arms) were studied. During median follow-up period of 880 days (range: 385–1545 days), CMAP amplitude increased (*p* = 0.02), and conduction block in the elbow segment recovered (from median 17% to 7%; *p* < 0.001). By contrast, SNAP amplitude did not change (*p* = 0.89). On needle EMG, spontaneous denervation activity diminished (*p* < 0.001), motor unit potential (MUP) amplitude increased (*p* < 0.001), and MUP recruitment remained unchanged (*p* = 0.43). **Conclusions:** Findings of the present study indicate that nerve function in chronic focal compression/entrapment neuropathies seems to improve mainly due to the resolution of the conduction block and collateral reinnervation. Contribution of nerve regeneration seems to be minor; the majority of axons lost in chronic focal neuropathies probably never recover. Further studies using quantitative methods are needed to validate present findings.

## 1. Introduction

Focal neuropathies are among the most common neurological disorders. According to epidemiological data from the province of Siena in Italy, we can calculate that, in Europe (population of 750 million), each year, around 2 million people are affected by median neuropathy at the wrist (i.e., carpal tunnel syndrome—CTS) [1]. The second most common focal neuropathy is ulnar neuropathy at the elbow (UNE), which, in Europe each year, affects around 150 thousand patients [2].

We reported previously outcomes of our cohort of patients with UNE after an average follow-up of almost three years. In the majority of these patients (83%), arm function improved at least moderately, and more than half of them (58%) reported major or complete recovery [3]. Theoretically, on the motor side, there are three main patho-physiological mechanisms of nerve function improvement: (1) reduction in conduction block, (2) collateral reinnervation, and (3) nerve regeneration (i.e., direct reinnervation). However, relative contributions of these mechanisms in recovery of function after chronic focal neuropathies are not well established.

In the present study, I tried to estimate the relative contributions of these three mechanisms in the recovery of function in patients with UNE due to external compression or entrapment. I compared findings of nerve conduction studies (NCSs) and qualitative concentric needle electromyography (EMG) at the time of presentation and at follow-up examination several years later. The contribution of the conduction block in the elbow segment was estimated by comparing amplitudes of the compound muscle action potentials (CMAPs) recorded from the abductor digiti minimi (ADM) muscle on ulnar nerve stimulation distal and proximal to the elbow. The contribution of muscle reinnervation was estimated by comparing CMAP amplitudes on ulnar nerve stimulation distal to the elbow at the time of presentation and at follow-up examination. For differentiation of collateral and direct muscle reinnervation, I compared findings of qualitative needle EMG of ADM on both examinations. In collateral reinnervation, the increase in motor unit potential (MUP) amplitude with unchanged MUP recruitment, and in direct reinnervation, the opposite would be expected. On the sensory side, nerve regeneration would present by increase in amplitude of the sensory nerve action potential (SNAP), which was recorded from the little finger.

## 2. Materials and Methods

### 2.1. Patients and Controls

I performed a post hoc analysis of the data obtained in a previously reported, prospectively recruited cohort of consecutive patients with clinical UNE diagnosis confirmed by electrodiagnostic (EDx) or ultrasonographic (US) examination [3,4]. Only patients with persistent UNE symptoms, and abnormalities on the first clinical neurologic examination that responded to our invitation to participate in a follow-up examination, were included in the present study. Other inclusion and exclusion criteria were as previously described [4]. The National Ethics Committee of Slovenia approved the study, and all participating patients provided written informed consent prior to the investigation [5].

### 2.2. History and Clinical Neurologic Examination

On the first examination, as well as during the follow-up examination, demographic and clinical data were collected using the patient’s history and a short questionnaire [6]. Hand paresthesia and weakness were graded as: 1—absent, 2—mild, 3—moderate, 4—severe, or 5—extreme. On clinical neurologic examination, ADM muscle wasting was graded as: 4—severe, 3—moderate, 2—mild, or 1—absent. We estimated ADM muscle strength using the extended Medical Research Council (MRC) scale [4], and graded light touch using cotton wool as: 1—normal, 2—reduced, or 3—absent. ADM strength was also measured in Newtons (N) using the Rotterdam Intrinsic Hand Myometer (RIHM) [5], and light touch was tested using Semmes-Weinstein monofilaments as: 1—normal, 2—diminished light touch, 3—diminished protective sensation, 4—loss of protective sensation, or 5—deep pressure sensation only [6]. Strength measurements were performed three times, using their average for further analyses.

We graded the clinical severity of UNE using a four point scale: (1) very mild UNE: only (sensory) symptoms; (2) mild UNE: (sensory) symptoms (as per very mild) + reduced sensation in the ulnar-innervated hand regions + normal motor function or mild ADM/FDI weakness (>4 MRC); (3) moderate UNE: sensation reduced (as per mild) + moderate ADM/FDI muscle weakness (4 MRC) + ADM/FDI muscle atrophy; (4) severe UNE: sensation reduced (as per mild) or absent + severe ADM/FDI muscle weakness (<4 MRC) + ADM/FDI muscle atrophy [7,8,9].

### 2.3. Electrodiagnostic Studies (EDx)

NCSs have been performed using standard EMG equipment (Nicolet Synergy, Natus Medical Incorporated, San Carlos, CA, USA). Short segment NCSs (SSNCSs) were performed by stimulating the ulnar nerve at the wrist and in 2-cm steps from 4 cm distal (D4) to 6 cm proximal (P6) to the medial epicondyle (ME) of the elbow. Ulnar CMAPs were recorded from the ADM muscle. Ulnar SNAPs were recorded from the little finger on stimulation 14 cm proximally at the wrist (i.e., antidromic technique). Concentric needle EMG of ADM muscle was performed. Abundance of spontaneous denervation activity (SDA) during muscle relaxation was described as: 0—absent, 1+—sparse, 2+—moderate, and 3+—dense. During stronger voluntary muscle activation amplitude (mV) of the highest reproducible MUPs, as well as MUP recruitment and interference pattern (IP) density (0—normal, 1−—mildly, 2−—moderately, 3−—severely reduced, 4—individual MUPs, 5—no MUP recruitment), were estimated.

During EDx studies, the electromyographer (SP) was blinded to patients clinical information (i.e., history and findings of the clinical neurologic examination), and on follow-up examination, he was also blinded on findings of the initial examination several years before. However, the electromyographer was aware of NCSs findings he obtained just before the concentric needle EMG study.

### 2.4. Statistics

Data were prepared in a standard spreadsheet (Excel, Microsoft, Redmond, WA, USA), and they were statistically analyzed using an on-line statistical calculator [10]. As all evaluated parameters were non-normally distributed, I calculated parameter median values and the 25th and 75th percentile limits. I also calculated the percentage of arms with reduced CMAP amplitudes at D4 (lower reference limit: 6.8 mV) and at P6 (lower reference limit: 6.6 mV), as well as reduced SNAP amplitudes recorded from the little finger (lower reference limit: 13 µV). For single comparisons of ordinal and non-normally distributed parameters, I used the Mann-Whitney U-test. I calculated correlations between differences in several parameters (i.e., CMAP amplitudes on D4 stimulation, conduction block, MUP amplitude, and MUP recruitment) and difference in ADM muscle strength measured by dynamometry between the first and follow up examination. Multiple linear regression analysis of the effect of these 4 independent parameters and the same dependent parameter were also calculated. In addition, correlation between the difference in SNAP amplitude, as well as the difference in monofilament testing between the first and follow up examination, were calculated. The significance level was set at α = 0.05 (two-sided).

## 3. Results

An amount of 170 patients (175 arms) were potentially eligible, having clinical diagnoses of UNE, confirmed by EDx or US examinations. Follow-up examination was performed in 111 of them (114 arms). These patients were, on average, 55 (range: 19–87) years old. The cohort included more men (65%), and the left arm was more often affected (65%) than the right. UNE was due to entrapment under the humeroulnar aponeurosis (HUA) in 47 arms (38 treated surgically), as well as due to external compression in the retrocondylar canal (RTC) in 61 arms (56 treated conservatively). In the remaining six arms, UNE localization was not clear.

The median time between the first and follow-up examination was 880 days (range: 385–1545 days). Except absent MUP parameters in two completely denervated ADM muscles on the first examination (none on the follow-up examination), there were no missing data.

During the follow-up period, all observed motor and sensory symptoms and signs significantly improved (Table 1). Patients reported complete disappearance of symptoms in thirty-three arms, marked improvement in thirty-three, and moderate improvement in twenty-six arms, as well as no change in fifteen arms and worsening symptoms in seven arms. Clinical severity of UNE was initially very mild in four arms, mild in fifty-seven arms, moderate in twenty arms, and severe in thirty-three arms, but these improved during the follow-up period to forty-three, forty-three, six, and twenty-two arms, respectively.

**Figure 1:**
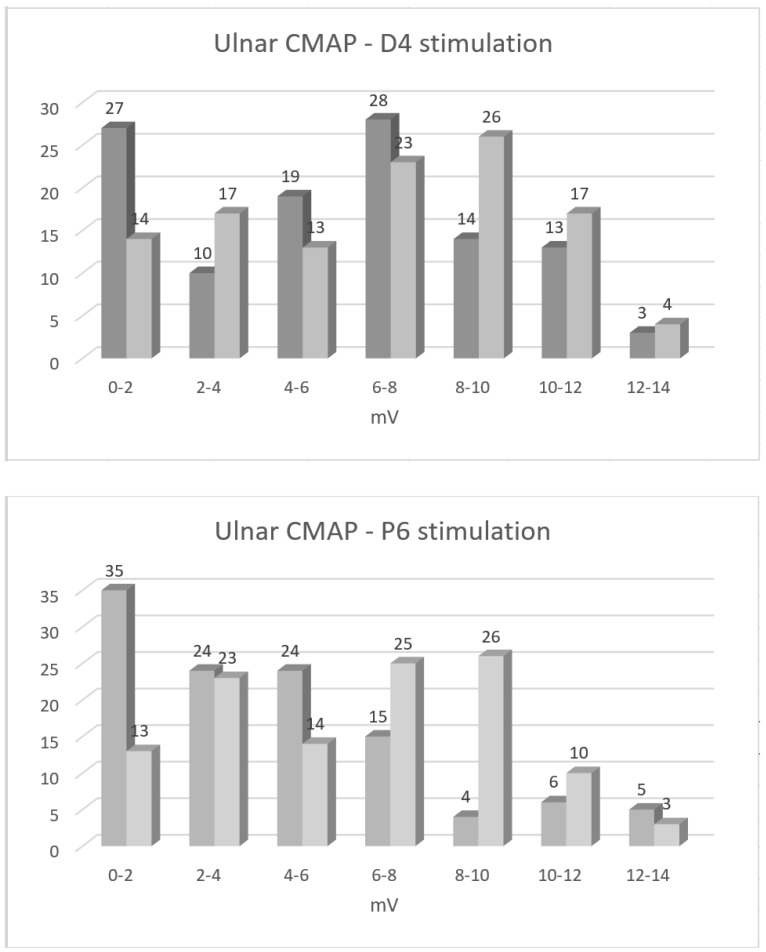
Amplitudes of compound muscle action potentials (CMAPs) on ulnar nerve stimulation at 4 cm distal (D4, above) and 6 cm proximal to the medial epicondyle (P6, below), and the recording from the abductor digiti minimi (ADM) muscle. Responses obtained on the first (dark gray) and on follow-up examination (light grey), being, on average, performed two and a half years later, are shown. The increase in CMAP amplitude on stimulation distal to the elbow (D4, above) is mainly due to collateral reinnervation. Increase in CMAP amplitude on stimulation proximal to the elbow (P6, below) occurs, in addition, due to resolution of the conduction block. The lower reference limit for this parameter is 6.3 mV.

During the follow-up period, median CMAP amplitude on ulnar nerve stimulation at D4 increased by 1.0 (25th perc.–75th perc.: −0.2–2.0) mV (*p* = 0.02), and, upon stimulation at P6, it decreased by 1.3 (0.3–3.2) mV (*p* < 0.001; Table 2). Conduction block in the elbow area diminished, on average, from 17% to 7% (*p* = 0.04, Figure 1). The percentage of arms with CMAP amplitudes below the lower reference limit reduced from 61% to 46% on D4, and they reduced from 81% to 47% upon P6 stimulation. By contrast, ulnar SNAP amplitudes did not change (*p* = 0.89; Table 2), and their proportion below the lower reference limit even increased during the follow-up period from 63% to 66%.

**Figure 2:**
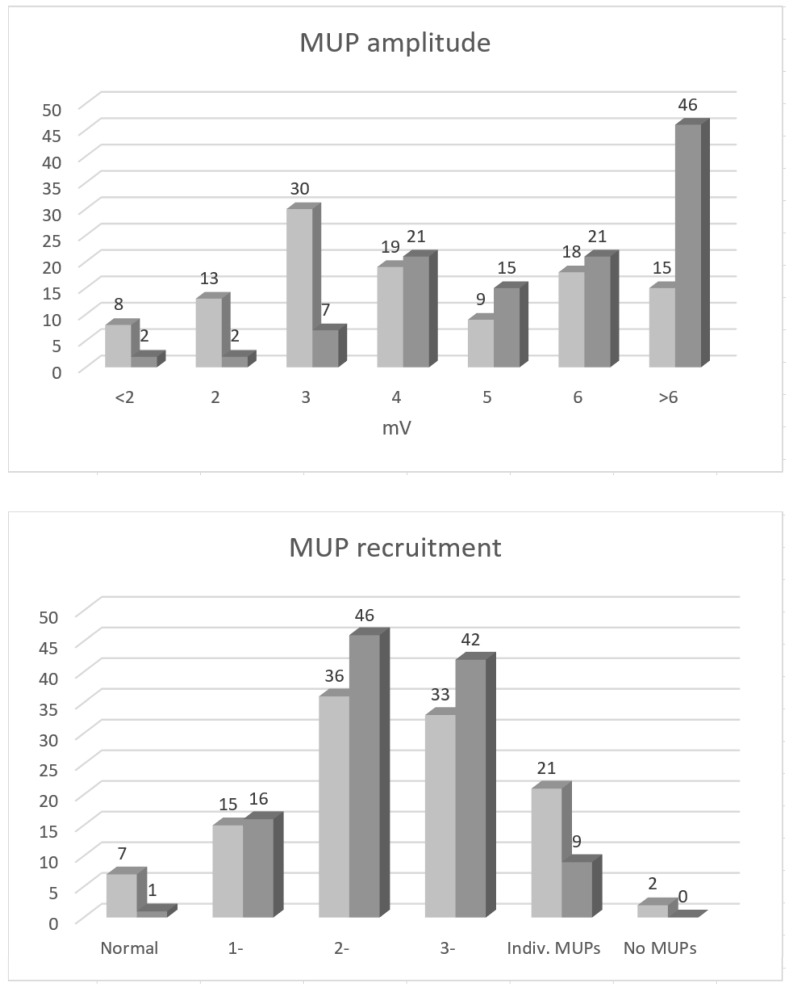
Amplitudes of motor unit potentials (MUPs, above) and MUP recruitment (below) in the abductor digiti minimi (ADM) muscle on qualitative concentric needle electromyography (EMG) on the first (light grey) and on follow-up examination (dark grey) performed, on average two and a half years later. Note the increase in MUP amplitudes and only the slight trend towards increased recruitment. Normal MUP amplitudes are of 2–3 mV.

Upon concentric needle EMG, SDA diminished significantly during the follow-up period (*p* < 0.001; Table 2). Upon qualitative MUP analysis, MUP amplitude increased, but MUP recruitment remained unchanged (Table 2, Figure 2). The same pattern of change in NCS and needle EMG parameters was also observed separately for arms with UNE due to entrapment, as well as due to external compression.

During the follow-up period, the change in all tested parameters, except MUP amplitude, demonstrated significant correlation with change in ADM dynamometry (Table 3). Upon multiple linear regression analysis, the change in three correlated parameters also had a significant effect on the ADM dynamometry change. These three parameters explained 25% of the variability in dynamometry change (R^2^ = 0.25). In our linear regression model, the relationship between the predicted and observed data was moderate (R = 0.50), and the model provided better fit than the model without independent variables (F = 12.25, *p* ≤ 0.001). Correlation between the change in SNAP amplitude, and the change in monofilament sensation, was not significant (Pearson correlation coefficient r = 0.03, *p* = 0.72).

During the follow-up period, CMAP amplitude on ulnar nerve stimulation at D4 increased >3 mV in 12 patients. Upon needle EMG in seven of them, the MUP amplitude also increased by >3 mV, with no major change in MUP recruitment. MUP recruitment improved >1 grade in seven arms: two were performed with no MUPs, four were performed with single MUPs, and one was performed with severely reduced MUP recruitment on the first needle EMG examination. The mechanism of improvement in MUP recruitment was, in two arms, related to the resolution of a severe conduction block (the improvements were 100% and 91%), and, in three arms, both reduction in conduction block (for 32%, 20%, and 16%) and reinnervation were observed, and, in the remaining two arms, reinnervation was observed (<10% reduction in conduction block).

## 4. Discussion

Findings of the present study suggest that, in UNE, the mechanisms of functional recovery are mainly the resolution of the conduction block, and, on the motor side, also collateral reinnervation. Based on the present findings, the contribution of nerve regeneration (i.e., direct reinnervation) seems to be less important. During an average follow-up period of two and a half years (minimal > one year), the conduction block in the ulnar nerve segment across the elbow was reduced by a median of 7% (Table 2, Figure 1). CMAP amplitude on stimulation distal to the elbow also increased significantly (median value 17%), pointing to reinnervation, either collateral or direct. However, concentric needle EMG findings suggests that this increase was mainly due to collateral reinnervation and not due to nerve regeneration. This conclusion is based on findings of significant increase in MUP amplitude, with unchanged MUP recruitment being observed during the follow-up period (Table 1, Figure 2). Unchanged MUP recruitment points to, more or less, unchanged number of motor units innervating ADM. Although not systematically studied, I did also not observe early reinnervating (i.e., nascent) MUPs in this, or in our other UNE cohorts studied at shorter intervals. The increase in CMAP amplitude on ulnar nerve stimulation distal to the elbow was, therefore, mainly due to MUP enlargement. In experiments, animals’ innervation ratio increased by up to three to five times [11], meaning that, after collateral reinnervation, MUPs can become at least three to five times larger.

These findings from the motor side were complemented by findings of unchanged SNAP amplitude during the follow-up period, speaking against significant nerve regeneration, also on the sensory side. There is no sensory equivalent of the collateral reinnervation on the motor side that could be detected using sensory NCSs, and, therefore, changes in SNAP amplitude directly measure changes in the number of sensory nerve fibers within the peripheral nerve. It is, however, interesting that I found correlation and significant effect on multiple linear regression of change in MUP recruitment, but not of maximal MUP amplitude on change in ADM strength (Table 2). The explanation for this might be that maximal MUP amplitudes are compensatory mechanisms, reflecting also the severity of nerve damage. Large MUPs, therefore, point to both efficient reinnervation and the severity of muscle denervation damage, thus first reducing, and, second, causing weakness. By contrast, MUP recruitment directly reveals the number of active MUPs that generate muscle strength.

The presented findings might seem surprising, as we would expect nerve regeneration to be much more important in functional recovery of compression/entrapment neuropathies. The explanation for this probably lies in limited optimal time for axonal regeneration, which is most efficient in the first four weeks after axonotmesis [12]. In cut nerves, all axons cross the suture line within 1 month [13], and only 5% of muscle mass is reinnervated after chronic denervation, as compared to immediate repair [11].

Initially, the main reason for this is progressive deterioration of Schwann cells in the intramuscular nerve sheaths [11]. To support the regenerating axons that sprout from the proximal nerve stump, Schwann cells and collagen fibers align in longitudinal “bands of Büngner” [14]. Many growth-associated genes are upregulated in denervated Schwann cells as they proliferate and switch from a myelinating to a growth-supportive phenotype. However, expression of these genes reaches its peak at seven days, and it declines to baseline levels within six months [15]. After the rapid early decline in regeneration ability caused by the Schwann cell denervation, later further deterioration is due to inability of the long-term denervated muscle to accept reinnervation [11,12]. Muscle size, weight, and contractile force all decline progressively with duration of chronic denervation [16]. After denervation, satellite cells divide and fuse to form multinucleated muscle fibers [17], but their limited number may preclude full recovery [16]. Not only are there fewer muscle fibers, but the size of the fibers is also reduced.

The large majority of arms with ulnar nerve entrapment was treated by surgical resection of HUA (80%), and the large majority of arms with external compression in RTC (92%) was treated conservatively [3]. I observed the same patterns of NCS and needle EMG parameter changes in HUA and RTC arms, as in the complete UNE cohort. Chronic nerve entrapment, as well as repeated habitual external compressions, usually persist for months or even years before diagnosis and eventual intervention. I would expect that, under such unwelcome conditions, bands of Büngner disintegrate and transform into the fibrous tissue, which further precludes growth of axon cones and nerve regeneration.

The main mechanism of long-term conduction block is demyelination [18], which often resolves after successful remyelination. According to the findings of the present study, remyelination is also one of two main mechanisms of UNE recovery. Even in asymptomatic subjects, ulnar nerve elbow segments demonstrate histological changes, indicative of previous demyelination and remyelination [19].

Lack of efficient direct reinnervation is probably not typical only of UNE, but also of other chronic focal neuropathies. In patients with median neuropathy at the wrist (causing CTS), lower thenar CMAP amplitude and larger MUP amplitudes on quantitative monopolar needle EMG studies were reported [20], pointing to collateral reinnervation as the mechanism of improvement. In CTS, no effective direct reinnervation occurs, in spite of a very short distance between the entrapment site and the thenar muscles. This also explains common clinical observation of persistent marked thenar muscle atrophy, even years after flexor retinaculum release. Similarly, in the present study, I also found that two thirds of severe UNE did not improve into the moderate category.

It is interesting that UNE patients report improved skin sensation in the ulnar area in spite of unchanged SNAP amplitudes (Table 1). I also did not find correlation between change in SNAP amplitude and change in sensation using monofilament during follow-up period. In addition to the improvement in the conduction block proximal to the assessed nerve segment, the mechanism of this sensory improvement may also involve central mechanisms.

The main limitation of the present study was the application of qualitative concentric needle EMG analysis. The problem may be sensitivity of qualitative EMG to detect minor differences occurring during nerve recovery. The present study findings, therefore, need to be regarded as preliminary. Quantitative motor unit number estimation (MUNE) and MUP analysis would be needed to validate them. A qualitative approach also introduced an element of subjectivity into analysis, although the same electromyographer performed all EMG examinations using consistent criteria. A large majority of EMG examinations are still performed using this approach. In addition, conclusions of the present needle EMG study were also based mainly on assessment of MUP amplitude and recruitment, which are the most robust qualitative EMG parameters. Furthermore, to obtain a representative MUP sample in severely neuropathic small hand muscles might be a problem, and about a third of arms in the present study had clinically severe UNE. The limitation of the study was also the electromyographer’s awareness of NCSs findings, which was unavoidable, as he performed NCSs just before needle EMG examination. Another limitation is exclusion of a significant proportion of UNE patients that did respond to our invitation for follow-up examination. However, it is not clear how this could change the main conclusions of the study.

The strength of the study is a rather large number of prospectively recruited patients and arms, using consistent inclusion and exclusion criteria. Prospective data on muscle reinnervation are limited, particularly in focal neuropathies. At the time of needle EMG examination, the electromyographer was also not aware of the patients’ clinical information. Another strength of the study is a long follow-up period, providing enough time for potential nerve regeneration to be completed.

## 5. Conclusions

During UNE recovery, ulnar CMAP amplitude increased, the conduction block resolved, and the SNAP amplitudes remained unchanged. Upon needle EMG, MUP amplitude increased, while MUP recruitment remained unchanged. These results suggest that, in UNE, nerve function improves mainly due to the resolution of the conduction block and collateral reinnervation, nerve regeneration being probably less important. However, further studies using quantitative methods would be needed to validate the present findings.

## Figures and Tables

**Table 1:** Comparison of neurological symptoms and signs in 114 arms with ulnar neuropathy at the elbow (UNE) on the first and follow-up examination.

	First Examination	Second Examination	Difference (2nd–1st)	*p*-Value
Symptoms				
Hand weakness	3 (1–4)	1 (1–2)	−1 (−2–0)	**<0.001**
Hand paresthesia	3 (3–4)	2 (1–2)	−1 (−2–(−1))	**<0.001**
Signs				
ADM atrophy	2 (1–3)	1 (1–2)	0 (−1–0)	**<0.001**
Extended MRC grading	5 (4–6)	7 (6–7)	1 (0–2)	**<0.001**
Dynamometry (N)	7.3 (3.7–10.0)	11.0 (6.5–13.7)	2.4 (0.2–4.9)	**<0.001**
Touch sensation	2 (2–2)	2 (1–2)	0 (−1–0)	**<0.001**
Monofilament testing	2 (2–3)	2 (1–2)	0 (0–1)	**<0.001**

Medians (25th percentile–75th percentile) are shown. Hand weakness and paresthesia were graded as: 1—absent, 2—mild, 3—moderate, 4—severe, or 5—extreme. The abductor digiti minimi (ADM) muscle wasting was graded as: 4—severe, 3—moderate, 2—mild, or 1—normal muscle bulk. ADM muscle strength was estimated using the extended Medical Research Council (MRC) scale (0–7) [4], and it was measured (N) using the Rotterdam Intrinsic Hand Myometer (RIHM) [5]. Touch sensation using cotton wool on the fifth digit was graded as: 1—normal, 2—reduced or 3—absent, and it was also graded using Semmes-Weinstein monofilaments as: 1—normal, 2—diminished light touch, 3—diminished protective sensation, 4—loss of protective sensation, or 5—deep pressure sensation only [6]. Significances (*p* < 0.05) are shown in bold.

**Table 2:** Comparison of nerve conduction studies (NCSs) and qualitative concentric needle EMG parameters in the abductor digiti minimi (ADM) muscle on the first and follow-up examination in 114 arms with ulnar neuropathy at the elbow (UNE).

	First Examination	Second Examination	Difference (2nd–1st)	*p*-Value
Nerve conduction studies				
CMAP amplitude D4 (mV)	6.0 (2.2–8.1)	7.1 (3.8–9.4)	1.0 (−0.2–2.0)	**0.02**
CMAP amplitude P6 (mV)	3.9 (1.5–6.1)	6.7 (3.2–8.7)	1.3 (0.3–3.2)	**<0.001**
Conduction block (%)	17 (7–43)	7 (3–11)	−7 (−31–3)	**0.04**
SNAP amplitude (µV)	6 (0–22)	5.5 (2–22)	0 (−6–2)	0.89
Needle electromyography				
Denervation activity	1 (0–1)	0 (0–0)	0 (−1–0)	**<0.001**
MUP amplitude (mV)	4 (3–6)	6 (4–8)	2 (0–4)	**<0.001**
MUP recruitment	5 (5–6)	5 (5–6)	0 (−1–1)	0.43

Medians (25th percentile–75th percentile) are shown. CMAP—compound muscle action potential; SNAP—sensory nerve action potential; MUP—motor unit potential; D4—stimulation 4 cm distal to medial epicondyle; P6—stimulation 6 cm proximal to medial epicondyle. Significances (*p* < 0.05) are shown in bold.

**Table 3:** Correlation and multiple linear regression of change in nerve conduction study (NCS) and needle electromyography (EMG) parameters, with change in the abductor digiti minimi (ADM) muscle dynamometry during the follow-up period in 114 arms with ulnar neuropathy at the elbow (UNE).

	Change in ADM Muscle Strength			
	Correlation		Regression	
	Pearson r	*p*-Value	Coefficient	*p*-Value
CMAP amplitude D4	0.28	**0.002**	0.60	**0.004**
Conduction block	0.36	**<0.001**	0.54	**0.001**
MUP amplitude	0.09	0.31	0.03 *	0.79 *
MUP recruitment	−0.35	**<0.001**	−0.85	**0.013**

CMAP—compound muscle action potential; D4—4 cm distal to medial epicondyle; MUP—motor unit potential. Regression was calculated using two step multiple linear regression analysis (R^2^ = 0.25, R = 0.50, F = 12.25, *p* ≤ 0.001). MUP amplitude was excluded in the second step of analysis; *—values obtained in the first step of analysis.

## Data Availability

The data presented in this study are available on request from the corresponding author. The data are not publicly available due to protection of patients’ privacy.
